# Participatory logic model for a precision child and youth mental health start-up: scoping review, case study, and lessons learned

**DOI:** 10.3389/frhs.2024.1405426

**Published:** 2024-10-17

**Authors:** Kathleen Pajer, Christina Honeywell, Heather Howley, Nicole Sheridan, Will Affleck, Ivan Terekhov, Dhenuka Radhakrishnan

**Affiliations:** ^1^ Department of Psychiatry, Children’s Hospital of Eastern Ontario (CHEO), Ottawa, ON, Canada; ^2^ CHEO Research Institute, Ottawa, ON, Canada; ^3^ Department of Psychiatry, University of Ottawa, Ottawa, ON, Canada; ^4^ Department of Paediatrics, Children's Hospital of Eastern Ontario (CHEO), Ottawa, ON, Canada; ^5^ Department of Pediatrics, University of Ottawa, Ottawa, ON, Canada

**Keywords:** precision mental health, precision health, children, youth, participatory logic model, implementation science, evaluation

## Abstract

**Background:**

The precision child and youth mental health (PCYMH) paradigm has great potential to transform CYMH care and research, but there are numerous concerns about feasibility, sustainablity, and equity. Implementation science and evaluation methodology, particularly participatory logic models created with stakeholders, may help catalyze PCYMH-driven system transformation. This paper aims to: (1) report results of a PCYMH logic model scoping review; (2) present a case study illustrating creation of a participatory logic model for a PCYMH start-up; and (3) share the final model plus lessons learned.

**Methods:**

*Phase 1: Preparation for the logic model* comprised several steps to develop a preliminary draft: scoping review of PCYMH logic models; two literature reviews (PCYMH and implementation science research); an environmental scan of our organization's PCYMH research; a gap analysis of our technological capability to support PCYMH research; and 57 stakeholder interviews assessing PCYMH perspectives and readiness. *Phase 2: Participatory creation of the logic model* integrated Phase 1 information into a draft from which the final logic model was completed through iterative stakeholder co-creation.

**Results:**

*Phase 1*: The scoping review identified 0 documents. The PCYMH literature review informed our Problem and Impact Statements. Reviewing implementation and evaluation literature resulted in selection of the Reach, Effectiveness, Adoption, Implementation, Maintenance (RE-AIM) and Behavior Change Wheel (BCW) frameworks to guide model development. Only 1.2% (5/414) of the organization's research projects involved PCYMH. Three technological infrastructure gaps were identified as barriers to developing PCYMH research. Stakeholder readiness interviews identified three themes that were incorporated into the draft. *Phase 2*: Eight co-creation cycles with 36 stakeholders representing 13 groups and a consensus decision-making process were used to produce the final participatory logic model.

**Conclusions:**

This is the first study to report the development of a participatory logic model for a PCYMH program, detailing involvement of stakeholders from initial planning stages to the final consensus-based product. We learned that creating a participatory logic model is time- and labour-intensive and requires a multi-disciplinary team, but the process produced stakeholder-program relationships that enabled us to quickly build and implement the PCYMH start-up. Our processes and final model can inform similar efforts at other sites.

## 1 Introduction

Child and youth mental health (CYMH) disorders are a significant public health problem. Worldwide, one out of seven 10–19- year-olds experiences a mental health disorder worldwide, accounting for 13% of the global burden of disease in this age group, whilst suicide is the fourth leading cause of death among 15–19 year-olds (1). Mental health care is insufficient to meet these needs in most parts of the world (2–5).

More concerning is that CYMH care, even when received, is not effective for many patients (6). Although the use of evidence-based CYMH psychotherapeutic treatments has grown, there has been no improvement, and for some of these treatments, a decline in effectiveness over time (7).

The same problems are increasingly reported with psychopharmacologic treatments. For example, a recent network meta-analysis of antidepressant medication efficacy in children and youth with depressive disorder reported “Most antidepressants may be associated with a “small and unimportant” reduction in depression symptoms on the CDRS-R scale (range 17 to 113) compared with placebo.” (8). The authors noted that there were likely sub-groups of patients for whom these medications were very effective, but others who received little symptom relief. Similarly, only 55%–60% of adolescents with anxiety disorders achieve remission with medication (9).

Transformation, not incremental change, in CYMH care and research is urgently needed (10). Precision health, which aims to tailor care to the individual's biological (e.g., genomic, metabolic, or neuroimaging biomarkers), lifestyle, psychological, and environmental characteristics holds great promise as a transformational paradigm (11–13). Each person's unique profile on these factors is matched with diagnosis, prognosis, or treatment based on research findings from stratification of large heterogeneous samples into homogenous sub-groups based on outcomes, not just symptoms. However, skepticism and concerns abound regarding the validity, feasibility, ethics, equity, and cost of precision child and youth mental health (PCYMH) care and research (14–16).

Moreover, transformation could easily fail in CYMH care systems that are already complex, i.e., containing multiple competing interdependent components and dysfunctional. To promote success and sustainability of transformation, it is imperative that leaders incorporate best practices from fields of implementation science and evaluation when developing PCYMH programs to improve care or research (17, 18). Furthermore, disseminating the program work they do is a vital component of systems transformation (19).

Logic models are the core of program implementation and evaluation. They are high-level graphic representations of the work to be done, although model structure and components vary by logic model type (20). They serve multiple purposes, e.g., a roadmap for program work and outcomes, a guide for evaluation of program success, or a visual tool to communicate with stakeholders and funders (20). Logic models are associated with more efficient and effective programs, including when used in CYMH settings (21–24).

Many program logic models are created by leaders and program planners or implementation experts, often with confirmatory stakeholder input obtained near the end of the process. In contrast, a participatory logic model engages stakeholder groups from the beginning and works with them throughout to co-create the model. This process increases engagement and outcome delivery, as well as contributes unique perspectives and ideas through the continuous collaboration on inputs, activities, outputs, and outcomes (25–28). Moreover, participatory logic models have been associated with greater effectiveness in outcomes in complex settings such as healthcare systems (29).

In 2022, the CHEO Research Institute (RI) in Ontario, Canada received donor funding to build the foundation for data-driven discovery and clinical PCYMH care through an 18-month start-up, called the PCYMH Initiative (PCYMHI). The RI is attached to CHEO, a 167-bed pediatric academic tertiary care hospital in Eastern Ontario. The RI and hospital are affiliated with the University of Ottawa.

To ensure optimal effectiveness and efficiency in a short period of time, we developed a participatory logic model to guide the PCYMHI build. The goals of this paper are to:

1. Report the results of a scoping review on PCYMH logic models.
2. Present a case study of the process involved in creating the PCYMHI participatory logic model.
3. Share the final model and lessons learned to aid other organizations in developing participatory PCYMH program logic models.

## 2 Methods

This case study of logic model development was done in two phases: Phase 1 - Preparation for logic model development and Phase 2 - Participatory creation of the final logic model. The project was run by a three-person core working group (KP, CH, and HH), intentionally small for efficiency and agility. However, co-authors participated in myriad tasks on the project and two evaluation consultants assisted with part of the work (SS and DH, see Acknowledgements). This project was deemed continuous quality improvement by the RI Research Ethics Board, not requiring Board review.

### 2.1 Phase 1: preparation for logic model development

This phase was organized around six questions to inform design of the preliminary draft of the model for Phase 2 work. Table 1 lists the six questions, methods used to answer them, and how the information was used. Each of the components of Phase 1 are briefly described in the following sections.

**Table 1:** Phase 1: questions, methods, and use in designing first draft of logic model.

Preparation questions	Methods	Use in logic model
1. Are there other PCYMH^a^ program logic models upon which we can build?	Scoping review about PCYMH logic models.	Search for PCYMH logic model to use as starting point.
2. What is the main problem we're trying to solve by building a PCYMH start-up?	Literature search about PCYMH.	Ensure efficient and effective delivery of outcomes to all relevant stakeholders within allotted time.
3. What is best implementation framework, theory, or model to inform creation of participatory logic model to meet our goals?	Literature search about implementation frameworks, theories, models.	Informed selection of framework to meet our goals.
4. What PCYMH work already exists at our site?	Environmental scan regarding our institutions’ PCYMH research or PCYMH clinical care services.	Identify what could be used as Resources; identify researchers and clinicians who can be champions if they are already doing PCYMH work; informed Activities and Outputs.
5. Is our Research Institute Informatics Core ready to support PCYMH research?	Informatics Core gap analysis.	If Informatics Core not ready, add building work into Inputs, Activities, and Outputs.
6.Who are our stakeholders, what are their perspectives on PCYMH, and how ready are they?	Semi-structured individual and group interviews, using combination of purposive and snowball sampling of organizational leaders, managers, researchers, providers, and external stakeholders.	Identify persons, services, departments, external organizations that can comprise Inputs or recipients of Outputs or Outcomes; obtain ideas for meaningful Activities, Outputs, and Outcomes; identify participants for the co-creation of logic model.

^a^ Precision Child and Youth Mental Health.

#### 2.1.1 Scoping review

We conducted a scoping review of the literature to investigate whether any other programs doing PCYMH care or research had disseminated information about their logic model (Table 1, question 1). Scoping reviews are designed to 1) identify and characterize studies to determine feasibility of a systematic review, 2) summarize how research has been conducted, 3) identify factors affecting findings, 4) delineate research gaps, and 5) present recommendations for researchers (30). We adhered to the Joanna Briggs Institute structure for scoping reviews (31) and followed the Preferred Reporting Items for Systematic Reviews and Meta-Analyses-Scoping Reviews (PRisMA-ScR) guidelines, as detailed in the PRisMA-ScR checklist (Supplementary Table S1) (32).

Our search strategy (Supplementary Table S2) used terms mapping onto constructs of age (e.g., “child”, “youth”, “adolescent”); mental health (e.g., “mental health”, “behavioral health”, “psychiatry”); precision health, (e.g., “precision health”, “precision medicine”); and “logic model”. Medical Subject Headings (MeSH terms) and keywords were incorporated into the strategy. Searches were conducted in PubMed and Embase from 1946 – March 3, 2023 and updated on August 15, 2023. Retrieved documents were combined to yield a dataset which has been shown to comprise academic publications, conferences, published abstracts, and books (33).

Eligibility criteria were established *a priori* to identify documents with the following characteristics: (1) description of a precision medicine or precision health logic model; (2) focus on mental health, psychiatry, or behavioural health; (3) focus on children or youth (0–23 years of age); and (4) written in English. There were no restrictions on study location, year of publication, or type of document. We planned to conduct blinded two-person abstract, title, and document reviews, followed by full text review for any documents meeting inclusion criteria. We then planned to synthesize the information from the final document data extraction. Disagreements were to be handled by consensus.

#### 2.1.2 Additional literature reviews

We conducted informal systematic reviews of two additional bodies of literature through PubMed searches (Table 1, questions 2 & 3). The first pertained to PCYMH, which informed the Problem and Impact Statements for the PCYMHI build. Such statements are critical for a program to determine its goals and shape the overall direction for a logic model (20).

The second review investigated the implementation science and evaluation research published to date to select a type of logic model (20) and an implementation framework, theory, or model (34). This selection process was guided by five questions suggested for this purpose by Lynch and colleagues (35): (1) Why? (your program aim); (2) Who? (individuals, groups, or organizations affected); (3) What? (what will be done & resources needed); (4) When? (timeframe for planning, implementation, & evaluation); and (5) How? (data sources). In synthesizing this literature to make our decisions, we prioritized institutional expectations that PCYMHI outcomes would be delivered within 18 months.

#### 2.1.3 Environmental scan and gap analysis

An environmental scan (Table 1, question 4) was conducted to determine the number of active PCYMH research projects at the CHEO RI using the institution's grant administration database (ROMEO, https://www.processpathways.com/). Similar information about PCYMH-related clinical services, improvement projects, or clinical research at the hospital was solicited through an online survey emailed to department leaders.

The Technical and Medical Directors of the RI Informatics Core assessed the extant hardware, software, cloud, and personnel infrastructure components in a gap analysis to determine the capacity and readiness to develop PCYMH research (Table 1, question 5). They benchmarked our infrastructure with comparable Canadian healthcare research institutes working towards similar goals.

#### 2.1.4 Stakeholder engagement

Identifying our stakeholders and understanding their views on PCYMH and readiness for this paradigm (Table 1, question 6) was crucial. Engaging them at the start of the model development process was essential for creating a participatory logic model. To that end, using a combination of purposive and snowball sampling strategies, the core working group invited 58 stakeholders to 18 virtual meetings.

Eleven stakeholder groups were represented: (1) Research Administrators, e.g., of the RI Informatics Core, the Clinical Research Unit; (2) PCYMH Researchers; (3) RI Leaders of relevant research programs, e.g., Genetics; (4) Outside Research Organizations; (5) the RI Healthcare Innovation Program; (6) Mental Health (MH) Clinical Researchers; (7) Clinicians; (8) Clinical Managers; (9) MH Program Administrators; (10) Youth MH Peer Support Group Leaders; and (11) Community Mental Health Agencies. The groups were selected because they comprised people or organizations impacted by PCYMHI, e.g., researchers and clinicians, or could contribute to PCYMHI as Inputs in the model, e.g., the RI Informatics Core. We did not include patients or caregivers in this phase, as we were focused on collecting internal and external organizational information.

The core working group used a semi-structured interview to gather information about PCYMH perceived benefits and concerns, suggestions for success, and participants’ “wish” lists for improving CYMH research or care. This method was selected to put people at ease and balance rapport-building with data collection in brief (45–60 min) virtual meetings with individuals or small groups (36).

Some of the first participants expressed discomfort in having the video recorded, so we did not tape meetings. Instead, we assigned a scribe to take detailed notes, for which we assured participants anonymity. The notes were then reviewed with participants before concluding each meeting, e.g., “So in summary, what we talked about was...”. Two co-authors (CH and WA) independently used inductive reasoning to informally identify themes in these notes and then used consensus to produce a final list of themes.

### 2.2 Phase 2: participatory creation of the final logic model

The core working group synthesized information from the literature reviews, environmental scan, and gap analysis, integrating this with the themes from the stakeholder interviews to develop a preliminary draft of the logic model. The consultants advised the working group on several revisions of this draft in preparation for stakeholder co-creation of a final logic model. The starting draft for the Phase 2 work was the result of several revisions based on discussion and consensus from the core working group and consultants.

Invitations were sent to 57 stakeholders across 13 groups to participate in the creation of the final logic model. Some of these groups had participated in Phase 1 stakeholder interviews, but new groups were included: youth, caregivers, RI Executive Leaders, Hospital Executive Leaders, PCYMHI-funded researchers, RI and Hospital Core Services, and the RI Foundation. These groups were added now because they would either be directly affected by, e.g., youth, caregivers, or need to contribute to the operationalization of PCYMHI, e.g., RI and Hospital Core Services such as Human Resources and Finance. Among the re-invited stakeholder groups, approximately 30% sent new representatives and the remainder were people who had been interviewed in Phase 1.

The participatory phase work was conducted in focus groups and through email dialogue. Four in-person groups were planned, but a COVID-19 surge with attendant restrictions and high clinical demands thwarted this plan, resulting in two in-person meetings, one virtual, and a transition to email conversations thereafter. The evaluation consultants facilitated the meetings, posing open-ended questions such as “Do you see yourself in this logic model?”, “What would you add or remove from any of the model's components?” and “How can we improve the model?”. Stakeholder input from each meeting or email discussion was incorporated into the subsequent draft. The model was declared complete when participants and the working group reached a consensus.

In addition to recording changes made in each iteration, we also gathered data to examine the content generated during the co-creation process. Our goal was to gain insights into potential issues that could influence behavior changes during implementation. To achieve this, we recorded the sessions, had them professionally transcribed, and combined the information with the email discussions. WA employed inductive reasoning to perform a thematic analysis on all the information, searching for recurring themes.

## 3 Results

### 3.1 Phase 1: preparation for logic model development

#### 3.1.1 Scoping review

**Figure 1:**
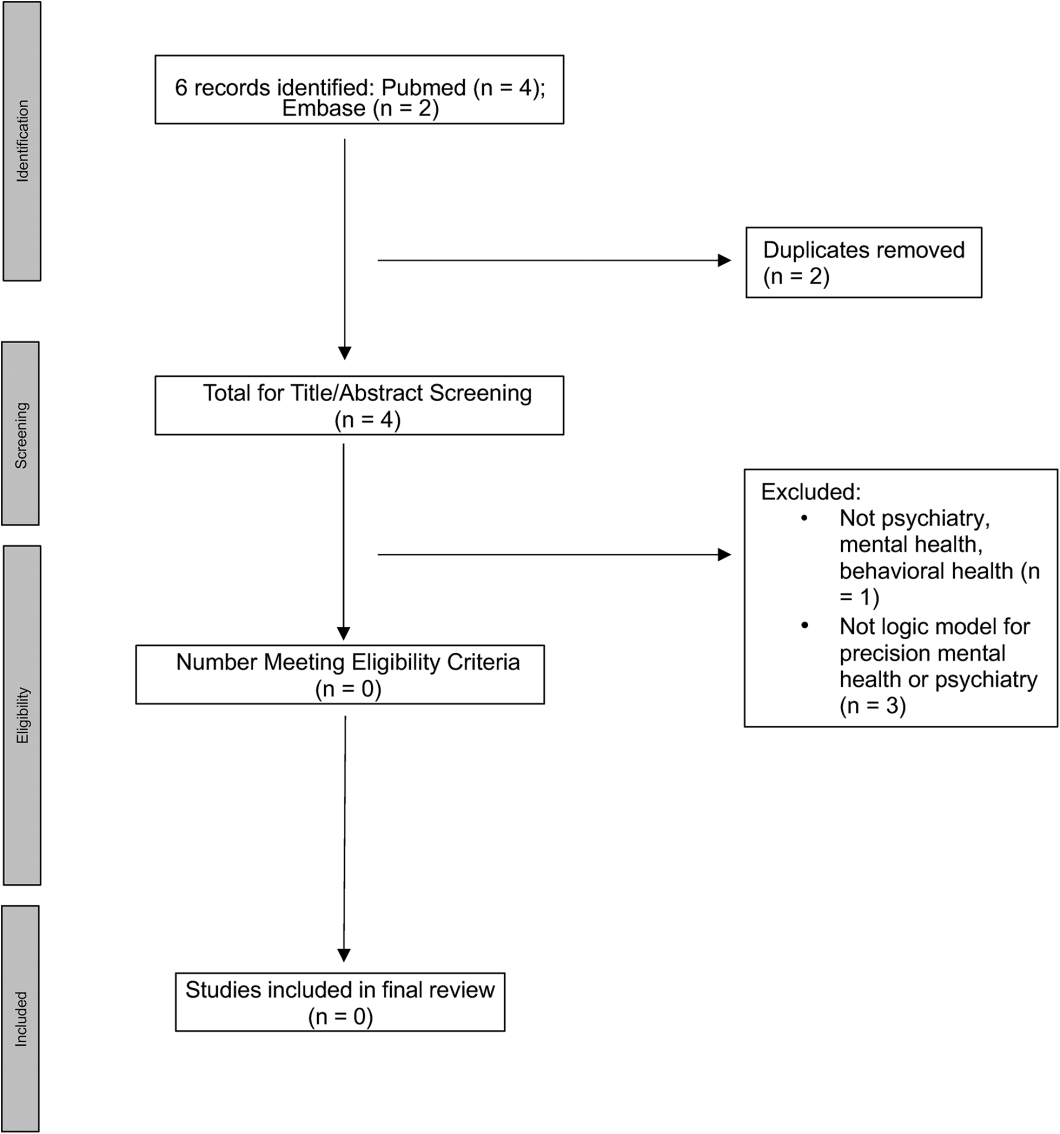
PRISMA diagram for relaxed criteria (without age limits) search.

Our original search strategy identified 0 documents. Therefore, we broadened the search by eliminating the age criterion and 6 documents were identified (see Figure 1). However, after removing 2 duplicates and conducting title and abstract screening of the remaining 4, we again found that 0 documents met inclusion criteria.

#### 3.1.2 Additional literature reviews

Based on our review of the PCYMH literature, we wrote the following PCYMHI Problem and Impact statements (20).

##### 3.1.2.1 PCYMHI problem statement

Child and youth mental health disorders are widespread and, if left untreated, can lead to long-term suffering, disability, and even mortality. Unfortunately, up to 50% of patients do not respond to recommended interventions due to inadequate treatment specificity for their needs. This lack of specificity stems from limitations in mental health research methodology, which hinders our ability to (a) understand the interaction between biological, psychosocial, and environmental factors in the development of mental health disorders and (b) discover effective treatments tailored to individual needs.

##### 3.1.2.2 PCYMHI impact statement

PCYMHI's overarching goal is to build a foundation for an efficient, effective, and sustainable PCYMH program producing ground-breaking research that is integrated with clinical care transformation to transform the mental health of Ontario's children and youth.

The review of implementation science and evaluation literature, combined with the requirement for quick delivery of outcomes, led us to select an outcomes type of logic model (20). To ensure a logic model relevant to program planning, implementation and evaluation, we chose the Reach, Effectiveness, Adoption, Implementation, and Maintenance (RE-AIM) framework (https://re-aim.org/). This decision was guided by criteria developed in a study of how > 200 implementation scientists choose implementation frameworks, theories, or models (37). The selection criteria most relevant to our needs were an implementation framework, theory, or model that: (1) would accommodate multiple analytic levels, e.g., individuals, teams, services; (2) had logical relationships between components; (3) provided utilization guidance; (4) had good empirical support; (5) was outcomes-focused; (6) provided a structure for program planning, implementation, and evaluation; (7) was easy for stakeholders to understand; and (8) included behavior change-driven Activities and Outputs.

In use since 1999 (38), the RE-AIM framework provides a planning, implementation, and evaluation structure that can lead to a value-based, pragmatic, outcomes-oriented logic models and programs in which implementation and evaluation are measured from the outset (39). Table 2 displays how each RE-AIM module helped shape components of our logic model.

**Table 2:** Phase 1: reach, effectiveness, adoption, implementation, maintenance (RE-AIM) framework-informed logic model development.

Framework sections	Framework section definition	How used in logic model
Reach	Absolute number, proportion, representativeness of individuals participating in start-up.	Helped identify participants for Activities, Outputs, Outcomes; also possible candidates for Inputs.
Effectiveness	Positive and negative start-up outcomes.	Definition and measurement plans for Outcomes.
Adoption	Absolute number, proportion, representativeness of participants using start-up during and at the end of funding.	Definition and measurement plans for who did Activities; who produced Outputs and 18-month Outcomes.
Implementation	Implementation is how well elements in a program are carried out, as well as costs and time needed to do so.	Program was being built as we were using it and this section informed our tracking of Outputs, Outcomes.
Maintenance	Program becomes institutionalized or part of routine organizational practices and policies; at individual level, it is long-term outcomes of the program.	Guided development of Long-term Outcomes (36-months) and Impact.

The RE-AIM framework met our criteria well, but given that we needed to change behavior in 18 months and that behaviour change is necessary for successful implementation (40), we wanted additional focus on this construct. The need to use more than one implementation framework, theory or model in planning a program and its logic model is not unusual and actually encouraged (41).

Therefore, to help us plan Activities grounded in behaviour science research, we also used the Behavior Change Wheel (BCW) framework (42, 43). Using a structured approach, the BCW posits that behaviour change is a consequence of interactions between Capability (physical and psychological), Opportunity (physical and social), and Motivation (automatic and reflective). To change behaviour of an individual or an organization, we need to operationalize the desired behaviour, define the actor(s), determine the best behavioural facilitator to target, i.e., Capability, Opportunity, Motivation, or some combination, determine the intervention types mapped to the behavioural facilitator targets, and plan intervention content that will match the best intervention types. It is important to optimize the likelihood that the intervention will succeed by evaluating it on Acceptability, Practicability, Effectiveness, Affordability, Side Effects, and Equity (APEASE).

We developed our desired organizational behaviors through the synthesis of the Phase 1 information, (44). Table 3 displays those desired organizational behaviours, who would change, the behavioural facilitator targets involved, the best type of interventions to be used and their definitions, and the interventions we created for the logic model Activities section. The APEASE checklist was used to ensure the activities were implementable.

**Table 3:** Phase 1: behavior change wheel (BCW) - desired organizational behaviours, behaviour targets, intervention types and definitions, corresponding logic model activities.

Desired behaviors by whom	Behaviour factor targets	Types of interventions & definitions	Corresponding logic model activities
PCYMHI leaders govern with key stakeholders	Opportunity motivation	Modelling: showing examples of behaviours for people to imitate.	Model transformative leadership structure & equity, diversity, and inclusion (EDI) ideas.
RI Informatics Core builds infrastructure facilitating PCYMH research and clinical care improvement	Opportunity motivation	Environmental Restructuring: constraining or promoting behaviour by shaping physical or social environment.	Restructure Environment to build big data computing, support artificial intelligence (AI), other PCYMH methods.
Legal and Privancy Offices write policies/procedures for private, secure data sharing	Capability opportunity motivation	Enabling: providing support to improve ability to change in ways not covered by other intervention types.	Enable secure data sharing within & between institutions.
Researchers conduct PCYMH studies; clinicians conduct PCYMH clinical improvement projects	Motivation	Incentivizing: changing attractiveness of a behaviour by increasing value of desired outcome or decreasing value of undesired one.	Incentivize PCYMH research & clinical quality improvement studies.
Researchers, clinicians, and persons with lived experience work together to conduct research or clinical improvement projects	Capability opportunity motivation	Educating: increasing knowledge and understanding by informing, explaining, showing, providing feedback. Training: increasing skills needed for a behaviour through repeated practice, feedback.	Educate & Train stakeholders in PCYMH research & care.
Organization members learn about PCYMH & executive leaders value and support PCYMH	Motivation	Persuading: using words, images to change how people feel about a behaviour to make it more or less attractive.	Persuade organization to learn about PCYMH & recognize initiative as a corporate project.

#### 3.1.3 Environmental scan and gap analysis

The combined output of the environmental scan of research revealed that only 1.2% (5/414) of active projects were about PCYMH. Similarly, there was only 1 clinical program using PCYMH methods.

The RI Informatics Core gap analysis identified three key components necessary for the data analytics requirements to facilitate PCYMH research: (1) a robust high-performance computing environment, (2) a dedicated data analytics and computing team skilled in business systems analysis, record extraction, AI data science, and biostatistics to work with electronic health record (EHR) and research data, and (3) a strong partnership between the RI and the hospital for PCYMH research and practice, which could challenge standard privacy and security practices through large-scale, multi-source data sharing or the use of EHR data. To enhance data access and facilitate new data sharing for local projects and external research collaborations, policies and standard operating procedures were needed. These policies would enable collaboration with hospital leadership, clinical care teams, the Research Ethics Board, legal counsel, privacy officers, and the EHR clinical operations team.

#### 3.1.4 Stakeholder engagement

Participation in the interviews was high, with 93% (54/58) of invitees attending interviews. Supplementary Table S3 lists the three stakeholder themes extracted about PCYMH readiness and perspectives: Potential Benefits of PCYMH; Barriers to PCYMH implementation; and Ethics Concerns. Participants were generally positive about the PCYMH opportunity, but were skeptical about the validity, feasibility, privacy and ethics. They also worried that the current EHR would be a major barrier because it was neither user-friendly nor set up for measurement-based care. Research concerns were the lack of adequate access to the EHR, lack of artificial intelligence (AI) and data science expertise, and an inadequate computing environment.

In addition, we received many suggestions for optimizing implementation success (Supplementary Table S4), which were grouped into three categories: (1) Engaging Patients and Families; (2) Communications About the PCYMH Program and Progress; and (3) Obtaining Buy-In from Clinicians, Researchers, and Staff.

### 3.2 Phase 2: participatory creation of the final logic model

Of 57 stakeholders invited to give feedback on the logic model draft, 63% (36/57) participated in one or more focus group sessions and email dialogues. Descriptions of the stakeholders are listed in Table 4. At least one representative from every stakeholder group participated. As mentioned in section 2.2, this list differed somewhat from the groups interviewed in the Phase 1, as the task of creating the model differed from determining PCYMH readiness. For example, this work included caregivers and youth, our internal executive sponsors, the CEO of the RI, and the hospital VP for Mental Health and Addictions.

**Table 4:** Phase 2: stakeholder description.

Groups represented	Number	Percentage of total	Percentage of invited
Clinician	7	12%	47%
Clinical manager	2	4%	40%
Mental health program leader	2	4%	100%
MH or CHEO researcher	2	4%	67%
Youth or caregiver	2	4%	67%
Research executive leader	1	2%	50%
Outside research organization	1	2%	100%
RI or CHEO core staff (HR, communications, patient experience, EDII office, medical records)	8	14%	80%
RI or hospital IS	4	7%	100%
Community agencies	1	2%	100%
PCYMH-funded researchers	3	5%	60%
Hospital executive administrators	1	2%	25%
Foundation	2	4%	100%

All stakeholder suggestions for each of the 8 iterations were incorporated immediately to create the next iteration until everyone was satisfied by consensus with the final product.

**Figure 2:**
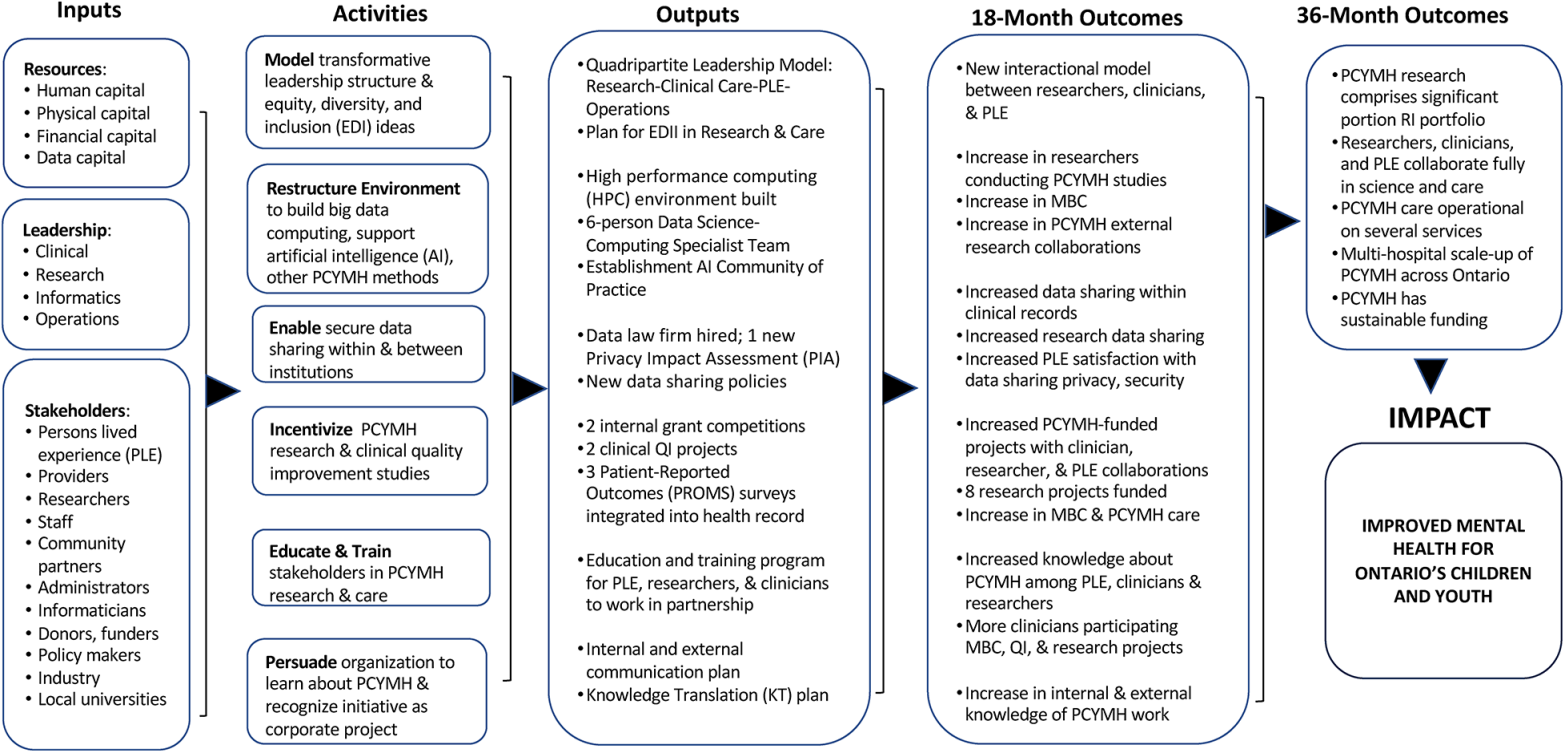
Final participatory precision child and youth mental health (PCYMH) start-up logic model.

Over the course of the eight versions, the logic model evolved to become more inclusive, sophisticated, and detailed, providing greater clarity to address stakeholder concerns. The final version prominently featured equity, diversity, and inclusion, along with enhanced robustness in outputs and outcomes related to EHR privacy and security, research data sharing, and computing and data analytic infrastructure (Figure 2). Additionally, a unique quadripartite care model that connects patients, clinicians, researchers, and operations personnel emerged from discussions. Table 5 shows two major categories of themes that arose in the analysis of the content of the co-creation process: Potential of PCYMH (3 sub-themes) and Concerns About PCYMH (eight sub-themes).

**Table 5:** Phase 2: participatory process stakeholder themes and quotes.

Themes	Sample quotes
I. PCYMH potential	
A. Improve treatment	“ I think for a lot of parents, they very much see that with medication in particular, it's a trial-and-error thing for getting it right... I, I too have lived experience. So, I’m very much both...someone who has lived that, trying medication, go off of medication...that's sort of part of my story as well.” Person with Lived Experience
B. Reassure patients and caregivers	"I feel like it's sort of an additional layer that would make me feel as a parent that my child is really being treated as an individual and here are all of the different ways that science now has to be able to treat a child as an individual.” Person with Lived Experience
C. Improve public health	“ The language [for the logic model work] ... is all downstream, treat sick kids language – appropriate but locked in the medical model trapped with treating sick kids and never preventing kids from getting sick in the first place. What about public health, health promotion, prevention – this is mostly missing... Can we consider the whole continuum of care (wellness through to illness? With equitable attention up-stream and down-stream)?” Clinical Researcher
II. PCYMH concerns	
D. Lack of infrastructure	"Right now we have no computer or research, within adequate research capacity to even allow the researchers and the computers to generate, you know, such a [PCYMH] profile for you.” Data Scientist
E. Proper inputs	“ What ____ [another participant] was saying is if we’re talking about the validity of the [data] input training, the providers that were talking about EHR, that should, I think, be captured about making it usable. Because it's not usable if they put the wrong things in the wrong places.” Clinical Researcher
F. Lack of trust	**"**Do they [patients] trust the institution? Do they trust the researchers with their data and how it's being used to, to inform research or, or, or any other type of decision-making?” Senior Hospital Administrator
G. Quality of data	"I wonder if there's another activity under Data... not related to the cleaning of the data, but if people don't trust the data. So, do we need to scrub data? Is that a thing?... Or like something about ensuring data quality or develop a process to ensure data quality. Maybe that's the activity?” Clinician
H. Stakeholder engagement	"If we rely on [patient and caregiver] surveys [for Patient-Reported Outcome Measures] for feedback [to measure outcomes], we are likely to get low response rates.” Clinical Researcher
I. Outcome measurability	Is the targeted impact that children, youth and families feel a decrease in “trail and error” therapy approaches? Or is it that there is a measurable decrease in trial and error approach? Either way, how would you measure that? I’m assuming the scope of impact at this point is within...patient population. IT Specialist
J. Lack of standardization	"We can build certain instruments into the EHR assuming that...you have the clinical buy-in of your group to, to actually capture the data discretely because oftentimes everybody has their own kind of style of documenting and preference and even...getting people to agree on the type of tools that you would use to evaluate patients would...take some time to actually get consensus...everyone has their preferred clinical scoring tools.” Research IT Specialist
K. Sensitivity of data	"One of the things that I’ve learned from ____[another participant], one of our senior scientists, is that we also need to make sure that people feel it's in their best interest to provide ethnicity [information] because there are a lot of people who feel that it may not be in their interest to raise their hand and say, ‘I’m of this ethnicity or this background.’ So it may just not be as simple as gathering data... just another thing to add a little more complexity...” Research Administrator

For example, all stakeholders were enthusiastic about PCYMH and excited about the downstream potential to predict an individual's future mental health problems and public health opportunities for prevention. Stakeholders were also excited by the potential of PCYMH to improve treatment, especially in the use of medications. By offering a more individualized approach, stakeholders felt that PCYMH would significantly improve the current trial-and-error approach to medication, which is both lengthy, and physically and emotionally taxing on patients and their families. A person with lived experience stated:

*"Well, I mean, I think for a lot of parents, they very much see that with medication in particular, it*’*s a trial-and-error thing for getting it right... And, I too have lived experience. So, I’m very much both, you know, someone who has lived that, you know, trying medication, go off of medication, you know, that*’*s sort of my, part of my story as well.”*

Common concerns were related to factors that could undermine the permanent PCYMH program or potentially do harm to patients or families. For example, if one type of PCYMH research relies on data from the EHR, concerns were raised about the quality of data. As one clinician stated:

*"I wonder if there*’*s another activity under Data... not related to the cleaning of the data, but if people don*’*t trust the data. So, do we need to scrub data? is that a thing?... Or like something about ensuring data quality or develop a process to ensure data quality. Maybe that*’*s the activity?” And a researcher later voiced, “ What ____ [another participant] was saying is if we’re talking about the validity of the [data] input training, the providers that were talking about EHR, that should, I think, be captured about making it usable. Because it*’*s not usable if they, you know, put the wrong things in the wrong places.”*

An example of concerns about avoiding harm with PCYMH was a theme about sensitivity of personal data, captured well by a comment made by a research administrator:

*"One of the things that I’ve learned from ____[another participant], one of our senior scientists, is that we also need to make sure that people feel it*’*s in their best interest to provide ethnicity [information] because there are a lot of people who feel that it may not be in their interest to raise their hand and say, ‘I’m of this ethnicity or this background.’ So it may just not be as simple as gathering data... just another thing to add a little more complexity...”*

Once the logic model was completed, we distributed it to stakeholders and used it to create a PCYMHI dashboard to begin tracking implementation and collecting data for the evaluation.

## 4 Discussion

The purpose of this project was to conduct a scoping review about PCYMH logic models, present the case study of our participatory logic model creation for the PCYMHI, and share our final model and lessons learned to assist others wanting to do the same at their sites.

We were surprised to find no existing logic models for PCYMH program planning in the scientific literature, meeting abstracts, or books. Initially, we thought this might be due to the novelty of focusing on child and youth precision psychiatry or mental health. To investigate further, we removed the age criterion and conducted another search, but still found no relevant documents.

The absence of other logic models in the precision mental health literature is concerning given the burgeoning number of new precision psychiatry/mental health programs and centers. Attention should be paid to implementation science and evaluation research in the development of these programs to maximize the likelihood of long-term success. Creating and using participatory logic models ensures that stakeholder perspectives remain the focus and that those affected and/or contributing to a program remain fully engaged (25, 26, 28). Moreover, PCYMH transformation of the mental health system of care and related research depends on cross-fertilization and communication between programs (19). Logic models are particularly valuable tools for sharing program information and although they may be organization-specific, can be readily adapted for other sites.

Our case study of creating a participatory logic model demonstrates the authentic, active involvement of researchers, clinicians, managers, administrators, community agencies, caregivers and patients, and various other external stakeholders. While stakeholder feedback may be setting-based, much of what stakeholders put into the model and what we shared can provide a starting point for other sites, e.g., suggestions for effective implementation and concerns about ethical issues, privacy, security, equity, and inclusion.

Although the scoping review did not identify logic models for precision psychiatry or mental health in any age group, we did find one participatory precision medicine logic model produced for a national genomic medicine program within the U.S. Veteran's Health Administration. It was informed by implementation science and stakeholder input collected during a one-day conference (45).

Despite not having a mental health focus, there were numerous similarities with our logic model. For example, their Activities included a Research category in which data sharing issues were listed, similar to our Activity of “Enable data sharing within and between institutions” (see Figure 2). They also created an Activities section for Precision Medicine Education, akin to our “Educate & Train stakeholders in PCYMH research & care”. And although they organized concepts and goals differently than we did, effects on ethics, equity, diversity, and inclusion were listed in their Key Impacts, which were included in our Outputs and Outcomes sections. These similarities offer some validation of our logic model for the broader program work needed for precision health programs.

### 4.1 Study limitations

There are three limitations to this case study. First, we were unable to conduct a full qualitative analysis of the preparatory work with stakeholders, as some were not comfortable being recorded. Although compensating by using a scribe who confirmed content of the notes taken at the end of sessions, it is possible that we may have missed some information. Second, only 63% (36/57) of invited stakeholders worked on Phase 2 due to increased clinical patient care and administrative demands during a concurrent surge of COVID-19, respiratory illnesses, and mental health emergencies. However, at least one representative of all stakeholder groups engaged in the process. Therefore, we believe that the likelihood of bias low, the final model being a a relatively accurate reflection of stakeholders’ ideas and opinions. Third, the logic model was created for a single pediatric tertiary care organization, which could reduce generalization to other settings or adult MH programs.

### 4.2 Lessons learned

#### 4.2.1 Lesson 1

Creating a participatory logic model is time-consuming. Ensure enough preparation time to conduct literature reviews, obtain baseline data for output and outcomes, and identify and fully engage all your stakeholders at the beginning of the process. Even more important is to allow enough time for the numerous iterations needed to produce a consensus model. Protected time or compensation for participation of stakeholders, including persons with lived experience may be necessary.

#### 4.2.2 Lesson 2

A multi-disciplinary team is necessary to create a participatory logic model. In our project, the process required scoping review expertise, content knowledge, skills in collecting interview data, facilitation experience, qualitative data analysis skills, and capability to integrate stakeholder information and feedback, results from literature reviews, and results of the environmental scan and gap analysis to produce the final logic model with the stakeholders.

#### 4.2.3 Lesson 3

The participatory logic model process significantly enhanced the implementation of PCYMHI. It fostered mutual trust and respect, quickly forming a cohesive team dynamic. This facilitated behavior change more smoothly than anticipated, as strong relationships were established between the PCYMHI leadership and over 60 individuals within the RI, hospital, and community.

## 5 Conclusions

PCYMH holds great promise for transforming mental health care and research, but there are numerous threats to the realization of that potential. Using a participatory logic model for program planning, guided by methods from the fields of implementation and evaluation science and disseminating the results could significantly increase the likelihood for PCYMH-driven system transformation.

## Data Availability

The original contributions presented in the study are included in the article/Supplementary Material, further inquiries can be directed to the corresponding author.
