# Supplementary material for: Participatory logic model for a precision child and youth mental health start-up: scoping review, case study, and lessons learned
Source: Front Health Serv. 2024 Oct 17;4:1405426. doi: 10.3389/frhs.2024.1405426 (PMC11524936; doi:10.3389/frhs.2024.1405426)
Supplement: Supplementary file 2 [file Table2.docx]

**Supplement 2** Scoping Review Search Strategy

| **Query #** | **Construct** | **PubMed (1946 – August 15, 2023)** | **Results** |
| --- | --- | --- | --- |
| 1 | precision medicine | "precision medicine"[All Fields] | 56,503 |
| 2 | precision health | "precision health"[All Fields] | 2468 |
| 3 |  | 1 OR 2 | 58,674 |
| 4 | mental health | "mental health"[MeSH Terms] OR ("mental"[All Fields] AND "health"[All Fields]) OR "mental health"[All Fields] | 498,862 |
| 5 | psychiatry | "psychiatrie"[All Fields] OR "psychiatries"[All Fields] OR "psychiatry"[MeSH Terms] OR "psychiatry"[All Fields] OR "psychiatry s"[All Fields] | 771,691 |
| 6 | behavioral health | ("behavior"[MeSH Terms] OR "behavior"[All Fields] OR "behavioral"[All Fields] OR "behavioural"[All Fields] OR "behavior s"[All Fields] OR "behaviorally"[All Fields] OR "behaviour"[All Fields] OR "behaviourally"[All Fields] OR "behaviours"[All Fields] OR "behaviors"[All Fields] OR "pattern"[All Fields] OR "pattern s"[All Fields] OR "patternability"[All Fields] OR "patternable"[All Fields] OR "patterned"[All Fields] OR "patterning"[All Fields] OR "patternings"[All Fields] OR "patterns"[All Fields]) AND ("health"[MeSH Terms] OR "health"[All Fields] OR "health s"[All Fields] OR "healthful"[All Fields] OR "healthfulness"[All Fields] OR "healths"[All Fields]) | 1,452,046 |
| 7 |  | 4 OR 5 OR 6 | 2,239,191 |
| 8 | logic model | "logic model"[All Fields] | 1,095 |
| 9 |  | 3 AND 7 AND 8 | 3 |
| 10 | children, adolescents, youth | "child"[MeSH Terms] OR "child"[All Fields] OR "children"[All Fields] OR "child s"[All Fields] OR "children s"[All Fields] OR "childrens"[All Fields] OR "childs"[All Fields] OR "adolescent"[MeSH Terms] OR "adolescent"[All Fields] OR "youth"[All Fields] OR "youths"[All Fields] OR "youth s"[All Fields] | 4,273,396 |
| 11 |  | 9 AND 10 | 0 |
| **Query #** |  | **Embase (1946 – August 15, 2023)** |  |
| 1 | precision medicine | precision medicine.af. | 44,888 |
| 2 | precision health | precision health.af. | 2,899 |
| 3 |  | 1 OR 2 | 47,787 |
| 4 | mental health | (('mental' and 'health) or 'mental health' or 'mental health').af. | 804,629 |
| 5 | psychiatry | (psychiatrie or psychiatries or psychiatry or psychiatry or psychiatry s).af. | 1,645,849 |
| 6 | behavioural health | ((behavior OR behavior OR behavioral OR behavioural OR behavior s OR behaviorally OR behaviour OR behaviourally OR behaviours OR behaviors OR pattern OR pattern s OR patternability OR patternable OR patterned OR patterning OR patternings OR patterns) AND (health OR health OR health s OR healthful OR healthfulness OR healths)).af | 1,338,079 |
| 7 |  | 4 OR 5 OR 6 | 3,788,557 |
| 8 | logic model | ‘logic model'.af. | 1,315 |
| 9 |  | 3 AND 7 AND 8 | 2 |
| 10 | children, adolescents, youth | ('child' or 'child' or 'children' or 'child s' or 'childrens' or 'childs' or 'adolescent' or 'adolescent' or 'youth' or 'youths' or 'youth s').af. | 4,831,037 |
| 11 |  | 9 AND 10 | 0 |
