# Supplementary material for: Participatory logic model for a precision child and youth mental health start-up: scoping review, case study, and lessons learned
Source: Front Health Serv. 2024 Oct 17;4:1405426. doi: 10.3389/frhs.2024.1405426 (PMC11524936; doi:10.3389/frhs.2024.1405426)
Supplement: Supplementary file 3 [file Table3.docx]

**Supplement 3** Phase 1: Preparation - Themes from Stakeholder Readiness Interviews

**Potential Benefits of PCYMH**

A. Opportunities for:

1. proactive identification of those at risk

2. early intervention

3. prevention of future mental health problems

4. guiding patients in self-help

5. tracking mental health outcomes and risk

6. impacting assessment and treatment

B. Reduction in:

1. harmful diagnostic and treatment siloes

2. clinician time, effort, and hassle reviewing charts

3. time patients spend repeating their backgrounds and struggles

**Barriers to PCYMH Implementation**

A. Electronic Health Record (EHR) technical or usage problems:

1. complexities of EHR which present different views to different providers

2. low use of EHR portal by patients and caregivers due to time, effort and perceived lack of relevance to care

B. Collecting or managing data:

1. lack of AI scientists and high-performance computing infrastructure to enable PCYMH research

2. lack of ability to compare or synthesize clinical information on patients for deep phenotyping across hospital services

C. Medicolegal issues:

1. concerns surrounding patient-completed questionnaire data entered in EHR

2. poor understanding of legal parameters for sharing of EHR and research data

**Ethics Concerns**

A. Regarding measurement-based care:

1. could replace the human connection so much needed by patients

2. would decrease job satisfaction in providers by reducing time spent with patients

B. Privacy of EHR data:

1. who has access

2. how it is used
