# Supplementary material for: Participatory logic model for a precision child and youth mental health start-up: scoping review, case study, and lessons learned
Source: Front Health Serv. 2024 Oct 17;4:1405426. doi: 10.3389/frhs.2024.1405426 (PMC11524936; doi:10.3389/frhs.2024.1405426)
Supplement: Supplementary file 4 [file Table4.docx]

**Supplement 4** Phase 1: Preparation - Tips for Success from Stakeholders

**Engaging Patients and Families**:

1. Co-design, using Research Institute (RI) and hospital family-youth partnership groups
2. Filling out questionnaires must be user-friendly, e.g., phone, rather than My Chart
3. Make My Chart more accessible and less confusing
4. Figure out how to manage questionnaire data entered by youth, caregivers, teachers
5. Ensure care and research extend to the community

**Communicating About Precision Child and Youth Initiative (PCYMHI)**:

1. Update communication methods with youth and caregivers, e.g., texts instead of emails
2. One size fits all won't work for effective communication - use multiple strategies
3. Hold gatherings and meetings in which you feed people
4. Reach out in person for some communications, not through emails
5. Use videos with emotionally-framed messages
6. Do communications within professional groups
7. Once PCYMHI is running, make it part of job descriptions and onboarding

**Obtaining Buy-In from Clinicians, Researchers, and Staff**:

1. Make electronic health record (EHR) more accessible for researchers and clinicians
2. Develop computer infrastructure to handle large amounts of data
3. Increase access to AI data analysis
4. Create PCYMHI Champions who will bring new information and progress to team members
5. Inform clinicians about caregiver and patient feedback on PCYMHI projects
6. Use what we have in EHR to help find patient characteristics, e.g., "flags", extant instruments
7. Create standardized ways to enter patients' social, lifestyle, and environmental data in EHR
8. PCYMHI innovations must make clinicians' work easier or show a positive impact on patients
9. Educate and train clinicians about PCYMHI innovations
10. Educate and train researchers and clinicians on new EHR access or on new data platforms
